# The Effect of Topical Cyclopentolate on Anterior Segment Parameters in Patients with Keratoconus

**DOI:** 10.4274/tjo.galenos.2019.51460

**Published:** 2020-03-05

**Authors:** Ahmet Kırgız, Sevil Karaman Erdur, Semih Çakmak, Funda Dikkaya, Rukiye Aydın

**Affiliations:** 1 University of Health Sciences Turkey, Beyoğlu Eye Training and Research Hospital, Clinic of Ophthalmology, İstanbul, Turkey; 2 İstanbul Medipol University Faculty of Medicine, Department of Ophthalmology, İstanbul, Turkey

**Keywords:** Cyclopentolate, keratoconus, cornea, corneal topography, anterior chamber

## Abstract

**Objectives:**

To investigate the effect of cycloplegia on anterior segment structures in keratoconus and forme fruste keratoconus patients using corneal topography.

**Materials and Methods:**

In this study, 40 patients with keratoconus (group 1), 40 patients with forme fruste keratoconus (group 2), and 40 healthy subjects (group 3) were evaluated prospectively. Flat keratometry (K) value (K1), steep K value (K2), mean K value (Kmean), maximum K value (Kmax), corneal astigmatism value, anterior chamber depth (ACD), symmetry index front, symmetry index back, thinnest corneal thickness, central corneal thickness and corneal volume were measured using Sirius topography before and after cycloplegia. Results were compared with one way ANOVA test.

**Results:**

The mean age of the participants was 24.4±6.2 years for group 1, 26.3±4.3 years for group 2 and 26.5±6.1 years for group 3. There was no difference between the groups with respect to mean age and gender (p>0.05). Mean K1 value was 45.54±2.43 diopters (D) before cycloplegia and 45.46±2.48 D after cycloplegia for group 1 (p=0.044). K1 value didn’t change significantly after cycloplegia for group 2 and 3 (p=0.275, p=0.371). There was no significant difference in K2 and Kmean values after cycloplegia for all groups (p>0.05). Kmax value decreased significantly after cycloplegia in group 1 (p=0.001), but the difference was not significant for group 2 and 3 (p=0.087, p=0.241). ACD increased significantly after cycloplegia in all groups (p=0.001).

**Conclusion:**

Cycloplegia causes corneal flattening only in manifest keratoconus patients, leading to an increase in ACD in all groups.

## Introduction

Keratoconus is characterized by progressive thinning of the cornea, corneal steepening, and irregular astigmatism.^1^ The mechanical and structural changes that take place in the corneas of patients with keratoconus lead to biomechanical weakening of the cornea. The factors that cause this include changes in collagen and extracellular matrix structure and keratocyte apoptosis.^2,3^

Although patients with manifest keratoconus can be diagnosed based on slit-lamp findings such as corneal steepening, Vogt lines, Fleischer ring due to iron deposition, and fractures in Bowman’s membrane that appear in advanced disease, corneal topography is currently the gold standard diagnostic method.^4^ Fellow eyes of patients with manifest keratoconus that do not display the typical findings of the disease and corneas that present suspicious topographic findings were described by Amsler^5^ as forme fruste keratoconus.

Cycloplegic agents are used in the diagnosis and treatment of ocular diseases by inducing relaxation in ciliary muscles. In doing so, they eliminate the accommodation needed to achieve a clear image and focus on objects at varying distances. In addition to refractive changes, preventing accommodation also leads to changes in anterior segment structures.^6,7,8,9,10^

Keratoconus typically begins in childhood, progresses into the 40s, and then halts progression.^1^ Accommodation is also at full capacity in this age group, and the effect of accommodation should not be overlooked when evaluating visual complaints.

The effects of cycloplegia on refractive defects and corneal and anterior segment structures are known.^6,7,8,9,10^ However, the effect of cycloplegia on biomechanically weak corneas, as in keratoconus patients, may differ from that seen in normal eyes. The effect of cycloplegia on keratometric values in eyes with keratoconus has only been investigated using an optical biometry device.^11^ In this study, we aimed to evaluate the effect of cycloplegia on anterior segment structures in eyes with clinical keratoconus and forme fruste keratoconus using corneal topography.

## Materials and Methods

This prospective and comparative study was conducted at the Beyoğlu Ophthalmic Training and Research Hospital of the Health Sciences University. Approval was obtained from the medical, surgical, and drug research Ethics Committee of Istanbul Medipol University prior to the study and the ethical standards set forth in the Declaration of Helsinki were followed throughout the study. The participants were informed about the nature of the study and possible results during the study. Verbal consent and signed informed consent forms were obtained from the participants.

A single eye of each patient was included in the study. If both eyes of a patient were eligible for the study, one eye was randomly selected. Forty eyes of 40 patients diagnosed with keratoconus were randomly selected and included in the study as group 1; 40 eyes of 40 subjects diagnosed with forme fruste keratoconus were included in group 2; and 40 eyes of 40 age- and sex-matched healthy volunteers who presented to our clinic for eye examination and general check-ups were included in group 3. Refractive error and ophthalmological examinations were performed by the same ophthalmologist for all patients. This examination included autorefractometer measurements, corrected and uncorrected visual acuity assessment, slit-lamp cornea and anterior segment examination, intraocular pressure measurements, and dilated fundus examination. Based on slit-lamp corneal examination and topographic findings, diagnoses of keratoconus and forme fruste keratoconus were made according to the criteria established in the Collaborative Longitudinal Evaluation of Keratoconus study.^1,12,13,14^

The study population included patients between the ages of 20 and 35 years with no history of ocular surgery or laser therapy and no other concurrent ocular pathology. Patients who had systemic disease, were pregnant or breastfeeding, used contact lenses, had a history of ocular trauma, displayed allergic or dry eye symptoms and findings, or had corneal scarring or nebulae were excluded from the study.

For all patients, refractive error measurements were made using an automatic kerato refractometer (ARK-1a, NIDEK Co., Japan). The spherical equivalent (SE) value to be used for statistical evaluation was calculated using the formula SE=spherical + cylindrical/2. Measurements of keratometric and anterior segment parameters were made with a Sirius device (Sirius tomography and corneal topography, CSO, Florence, Italy). Cycloplegia was achieved by instilling 1% cyclopentolate hydrochloride (Sikloplejin, Abdi Ibrahim) 3 times at 5-minute intervals. Automatic kerato refractometer and Sirius topography measurements were repeated 45 minutes after the last drop. We evaluated post-cycloplegia changes in the following parameters: flat keratometry (K) value (K1), steep K value (K2), mean K value (Kmean), maximum K value (Kmax), corneal astigmatism value, anterior chamber depth (ACD), symmetry index front (SIf), symmetry index back (SIb), thinnest corneal thickness (TCT), central corneal thickness (CCT), and corneal volume.

### Statistical Analysis

IBM SPSS for Windows version 22.0 statistical software was used for statistical analysis. The Shapiro-Wilk test was used to evaluate the normality of data distributions in the groups. Categorical variables were compared using chi-square test. Continuous variables were compared between groups with one-way variance of analysis (ANOVA) with Bonferroni post hoc test. Paired-samples t-test was used for within-group comparisons of continuous variables. Statistical significance was accepted as p<0.05.

## Results

Of the 120 patients included in the study, 68 were men and 52 were women. Mean age was 24.4±6.2 years in group 1 (20-33 years), 26.3±4.3 years in group 2 (20-34 years), and 26.5±6.1 years in group 3 (20-35 years). There was no significant difference between the groups in terms of age or sex (p>0.05).

The mean keratometric values (K1, K2, Kmean, and Kmax), SE value, corneal astigmatism value, ACD value, SIf and SIb values, TCT and CCT values, and corneal volume values of the groups before and after cycloplegia are shown in Tables 1, 2, and 3. There was a statistically significant increase in ACD and a significant hypermetropic change in SE value after cycloplegia in all groups (p=0.001).

When the keratometric values were analyzed, mean K1 value in group 1 was 45.54±2.43 diopters (D) before cycloplegia and 45.46±2.48 D after cycloplegia, which was a statistically significant difference (p=0.044). No significant change was observed in K1 after cycloplegia in group 2 or 3 (p=0.275, p=0.371, respectively). None of the groups showed a significant change in K2 or Kmean values after cycloplegia (p>0.05 for all). While there was a significant decrease in Kmax values after cycloplegia in group 1 (p=0.001), the decrease in group 2 was not statistically significant (p=0.087). The change in Kmax was also nonsignificant in group 3 (p=0.241). TCT and CCT values increased significantly in group 1 (p=0.028, p=0.016, respectively), but there was no significant change in group 2 or 3 (p>0.05 for both).

## Discussion

In this study evaluating the effect of cycloplegia on anterior segment structures in eyes with keratoconus and forme fruste keratoconus and a control group, we observed a significant decrease in K1 and Kmax values in the clinical keratoconus group after cycloplegia. This decrease demonstrates that cycloplegia causes corneal flattening in eyes with keratoconus.

Previous studies that examined the effects of accommodation on the refractivity of the cornea have had varying results. While Cheng et al.^7^ detected corneal flattening after instilling 0.04% tropicamide, Yasuda et al.^15^ reported an increase in corneal power after inducing ciliary muscle contraction with 4% pilocarpine. It is believed that ciliary muscle contraction causes corneal steepening by acting on the peripheral cornea via the scleral spur, whereas after cycloplegia this effect is eliminated and the cornea flattens.^7^ Contrary to these studies, others have indicated that accommodation has no effect on corneal refractivity.^16,17^ 

*Ex vivo* studies have shown that biomechanically, eyes with keratoconus have a low Young’s modulus, which also explains the pathophysiology of the disease.^18^ In addition, there are also studies demonstrating *in vivo* that corneal hysteresis, which is a marker of corneal biomechanics, is lower in eyes with keratoconus compared to normal eyes.^19,20^ We believe that relaxation of the ciliary muscles after cycloplegia causes corneal flattening through its effect on the biomechanically weak keratoconic cornea. Indeed, Polat and Gündüz^11^ similarly found a significant decrease in mean K1 and K2 values after cycloplegia in their study examining the effect of cycloplegia on keratoconic eyes with optical biometry. There are also studies that evaluated the corneal biomechanics of eyes with suspected keratoconus. Although some of these studies demonstrated low biomechanical properties similar to eyes with manifest keratoconus^21^, others did not show this effect.^22^ The lack of significant changes in the keratometric values of the control group and eyes with suspected keratoconus after cycloplegia in our study may result from their corneas being biomechanically stronger.

In the present study, Kmax and K1 values in eyes with keratoconus flattened by 0.35 D and 0.08 D, respectively, after cycloplegia. Although this change was found to be statistically significant, we believe it may be overlooked in the diagnosis of patients with manifest keratoconus. In addition, we think that pre-cycloplegia evaluation is more appropriate when evaluating keratoconus progression and especially in the follow-up of patients undergoing corneal cross-linking treatment. 

In their study of healthy volunteers, Bagheri et al.^8^ observed a significant increase in central and paracentral corneal thickness with cycloplegia. Chen et al.^23^ showed that corneal thickness increased following the administration of benzalkonium chloride (BAC) in their rabbit study and suggested that corneal edema occurred as a result of epithelial and endothelial damage caused by topical BAC.^23^ In the present study, our findings that increases in TCT and CCT were not statistically significant in the suspected keratoconus or control groups (p>0.05) but were significant in the keratoconus group (p<0.05) may be due to the corneas being thinner in keratoconus. The increase in corneal thickness that occurs due to corneal edema may have caused a significant difference in the thinner keratoconic corneas.

Many previous studies on healthy subjects and keratoconic patients have demonstrated an increase in ACD with cycloplegia.^6,7,8,9,10,11,24^ This is due to the decrease in lens thickness that occurs as the ciliary muscles relax and lens zonules tighten with cycloplegia. There is also posterior displacement of the lens. Increased ACD is expected as a result. In our study, we showed that in addition to healthy subjects and patients with keratoconus, this change in ACD after cycloplegia was also present in eyes with forme fruste keratoconus. In addition, Türkçüoglu et al.^10^ demonstrated that in eyes in which pupil dilation was induced with phenylephrine alone and accommodation was unaffected, ACD was also unaffected.

One of the methods used to improve vision in keratoconic patients is the placement of posterior chamber phakic intraocular lenses.^25^ ACD measurement is important when deciding to implant an aphakic intraocular lens.^26^ In addition, new generation formulas take into account ACD when calculating intraocular lens power.^27^ It has been reported that 42% of postoperative refractive errors are due to incorrect measurement of ACD.^28^ Therefore, measurements should be made before cycloplegia in patients with keratoconus who will undergo phakic intraocular lens implantation.

### Study Limitations

Limitations of our study are that we did not repeat measurements while stimulating accommodation or measure axial length and relative lens position.

## Conclusion

In conclusion, the present study demonstrated corneal flattening after cycloplegia in patients with manifest keratoconus, as well as a positive shift in SE and an increase in ACD with cycloplegia in all groups. This should be kept in mind during refractive error examination, disease progression follow-up, and contact lens and phakic intraocular lens applications in keratoconus patients.

## Figures and Tables

**Table 1:**
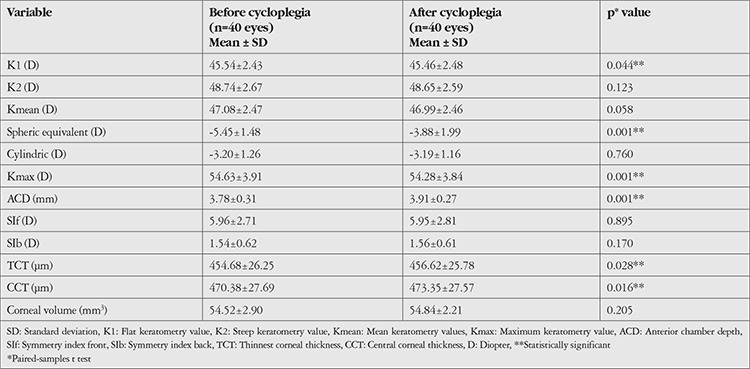
Topographic values before and after cycloplegia in the keratoconus group

**Table 2:**
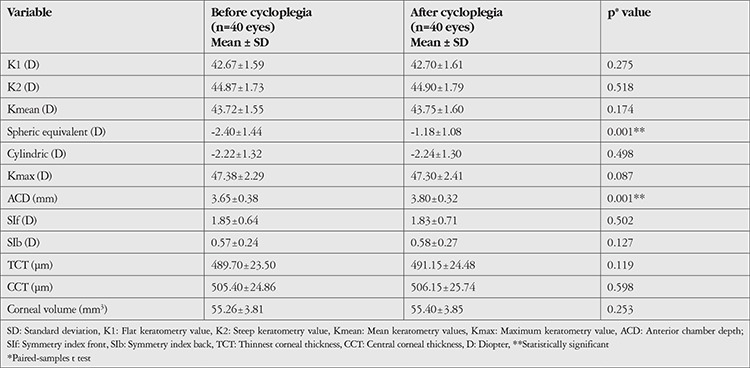
Topographic values before and after cycloplegia in the forme fruste keratoconus group

**Table 3:**
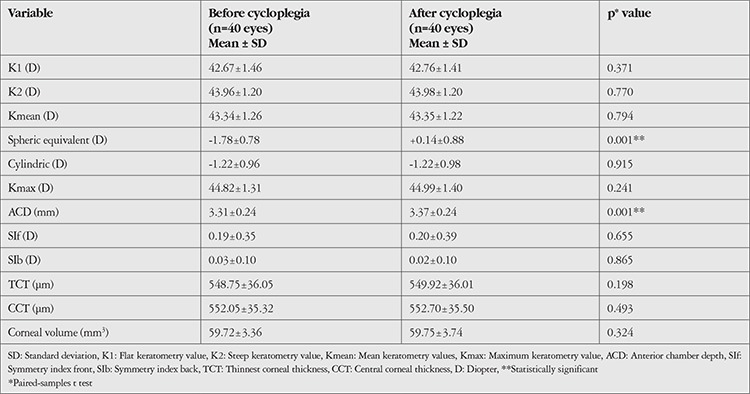
Topographic values before and after cycloplegia in the control group
